# Prevalence trends and risk factors for allergic rhinoconjunctivitis, asthma and eczema in the UK

**DOI:** 10.1186/s13223-025-00975-2

**Published:** 2025-07-07

**Authors:** Lavanya Diwakar, Anuradhaa Subramanian, Divya K. Shah, Sumithra Subramaniam, Victoria S. Pelly, Sheila Greenfield, David Moore, Krishnarajah Nirantharakumar

**Affiliations:** 1 Institute of Applied Health Research, University of Birmingham, B15 2TT Birmingham, UK; 2 Dept of Immunology, University Hospital of North Midlands, Stoke- on -Trent, ST4 6QG UK; 3 Wellcome Trust, London, NW1 2BE UK; 4 Midlands Health Data Research UK, Birmingham, UK

**Keywords:** (6 max): allergic rhinoconjunctivitis, Asthma, Eczema, Allergy, Prevalence, Risk factors

## Abstract

**Background:**

Allergic rhinoconjunctivitis (ARC), asthma and eczema carry a substantial morbidity. These conditions often co-exist within the same individual and their prevalence can differ based on age, ethnicity and gender.

**Objectives:**

Using a UK primary care database, we estimated the trends in prevalence over the last decade for ARC, asthma and eczema and associated risk factors.

**Methods:**

Longitudinal cohort analysis of the health improvement (THIN) database between 1st Jan 2010 and 1st Jan 2019. Logistic regression analysis was used to explore risk factors for diagnosis of these conditions.

**Results:**

An average of 4.17 million records per year were analysed, 19.4% were children and 49.75% were male. There was an increase in prevalence of ARC, asthma and eczema amongst adults during the study period, whereas ARC and asthma prevalence amongst children has fallen. By 2018, 1:8 adults and 1:14 children had ARC; asthma was diagnosed in 1:7 adults and 1:10 children whereas eczema was diagnosed in 1:6 adults and 1:4 children respectively. There were regional discrepancies in allergy prevalence across the UK. Caucasians generally had the highest rates of asthma and lower rates of ARC compared with other ethnic groups. Having other allergies substantially increases the odds of having asthma, eczema and ARC.

**Conclusion:**

The population burden of ARC, asthma and eczema in the UK is substantial. These conditions are often associated with other allergies and can, therefore, be complex to manage. These data support calls for improvement of pathways of care for allergy patients in the UK.

**Supplementary Information:**

The online version contains supplementary material available at 10.1186/s13223-025-00975-2.

## Background

It is clear that the incidence and prevalence of allergies worldwide is increasing [1]. Data from the International Study of Allergies and Asthma in Childhood (ISAAC), a cross-sectional epidemiological study, suggests that there has been a rise in the prevalence of parent-reported asthma, rhinitis and eczema amongst 6–7 year old and 13–14 year old children globally [2]. Similar trends in food allergy have been reported from longitudinal follow up of birth cohorts from the West Midlands and, more recently, from across Europe [3, 4].

Allergic rhinoconjunctivitis (ARC), asthma and eczema are common allergic conditions that carry significant morbidity. ARC has been associated with depression [5] shown to affect academic performance [6] reduce work place productivity [7] and exert a considerable financial burden on society [8]. Eczema is associated with significant reduction in the quality of life of affected individuals and carers [9] depression and anxiety [10] and also exerts a considerable financial pressure on individuals and society [11]. Asthma continues to be a condition associated with high morbidity and mortality world-wide [12]. It too has a substantial impact on the global health economy [12–14].

There is an epidemiological association between ARC, asthma and eczema. The so called ‘allergic march’ describes the strong risk of developing asthma and ARC in children with eczema [15]. While the underlying mechanisms related to this phenomenon are complex and not fully understood, genome wide association studies have identified certain genetic loci that may provide some explanation [16]. Mutations in filaggrin, a protein useful in maintaining epithelial barrier function, are strongly associated with eczema as well as asthma and ARC [17]. There are important clinical implications to this association as well. Asthmatics with ARC are known to have more exacerbations requiring higher doses of reliever medication when ARC symptoms worsen (e.g. during summer in hay fever sufferers). Not only do individuals with severe eczema carry a higher risk of having concomitant asthma and ARC, but they are also at higher risk of severe presentations of these conditions [18].

In the UK, almost all the daily activity of the UK National Health Service (NHS) is documented using bespoke data collection systems. Some of these national health data are made available for research. The advantage of investigating such national health datasets is that they are representative of the population of the country as a whole and the results obtained can be applicable to all regions of the country. The data can be used to assess the scale of the problem, and to identify areas where investments can be targeted to obtain maximal improvement in patient care.

There are some previously published data on allergy activity obtained from UK primary care databases showing that allergies affect approximately 39% of children and 30% of adults respectively [19]. About 4.5% of the population were estimated to have multiple allergic disorders in 2005 [20]. In Scotland, the life time prevalence estimates for eczema, asthma and ARC were 10%, 4.8% and 6% resepectively. A recent publication using primary care data showed that food allergy prevalence has almost tripled between 2008 and 2018 in the UK [21]. A study using multiple datasources between 1996 and 2004 found high rates of eczema, asthma and ARC but there was a suggestion that the prevalence rates of these conditions may be stabilising [22]. Recent data on the prevalence of these conditions and a broader exploration of demographic and clinical factors associated with their diagnosis are lacking.

Our study was aimed at estimating the prevalence trends and the associated risk factors for eczema, asthma and ARC in the UK using the Health Improvement Network (THIN) database, a database holding routinely collected data from primary care consultations in the UK. GPs serve as gatekeepers within the UK NHS and almost all UK population is registered with a GP. Therefore, examination of routinely collected UK primary care data can provide fairly robust population estimates of allergy prevalence [23].

## Methods

### Data source

THIN currently includes electronic patient records from 808 primary care practices across the UK, covering a total of over 16 million patients (about 3.6 million patients who are currently registered in these practices), who represent about 5.7% of the whole UK population [24]. These patients are generalisable to the UK population by age, gender, medical conditions and death rates [25]. More details about THIN and other routinely collected UK databases are available elsewhere [26].

Participating practices use Vision^®^ software to maintain patient records and issue prescriptions. Entries into THIN reflect patient demand for primary health care services; clinical entries into the database may be made by GPs or other authorised practice staff. Medical conditions are identified in the THIN database using Read codes [27]. The Read codes which were used in this study are shown in additional data provided with this paper (S1).

### Study population

General practices were considered eligible one year after reporting acceptable mortality rates [28] and one year after initiating use of the Vision^®^ system for documenting patient medical records. Participants were considered eligible one year after registration with an eligible general practice.

We obtained patient records of all eligible individuals registered with an eligible general practice providing data to THIN between 1st January 2010 and 1st January 2019.

### Statistical analysis

Statistical analysis was performed using Stata16 software.

### Prevalence estimation

To estimate point prevalence every year from 2010 to 2019, all eligible individuals within the database at the start of the index year were included in the denominator cohort.

Annual point prevalence of each of the three allergic conditions (ARC, asthma and eczema) at the start of each year was calculated by dividing the number of eligible individuals with a Read code diagnosis for that condition prior to a specific year (see additional data) by the number of eligible individuals at the start of that year. Prevalence estimates were also obtained separately for adults (≥ 18 years old) and children (< 18 years old); males and females.

### Regression analysis

In a cross-sectional snapshot of patients eligible on 1st of January 2018, logistic regression analysis was carried out among adults and children separately to obtain adjusted odds ratios and their corresponding 95% confidence intervals (CI) for the outcome ARC, asthma and eczema. Age categories, sex, ethnicity, region and presence of other allergic conditions were considered as independent variables in the model. The odds were separately estimated for children and adults. Age was stratified into subgroups of 10-year age bands [among adults: (1) 18–29 years, (4) 30–39 years, (5) 40–49 years, (6) ≥ 50 years; among children: (1) < 10 years, (2) 10–17 years]. Ethnicity of patients were categorised into one of the following: (1) Caucasian, (2) Black Afro-Caribbean, (3) South Asian, (4) Mixed race, (5) Other ethnic minorities (which included Chinese, Middle-Eastern and ‘other’), and a separate ‘not-specificed’ category was considered for patients without a record of their ethnicity. Region of the practice’s patients are registered with were stratified as: (1) East of England, (2) London, (3) North-West of England, (4) Northern Ireland, (5) Scotland, (6) South Central England, (7) South-East coast of England, (8) South-West of England, (9) Wales, (10) West Midlands, (11) Yorkshire & Humber. In addition to the three main allergic conditions of interest (ARC, asthma and eczema), other allergies including food allergy, drug allergy, urticaria and anaphylaxis were considered as independent variables.

## Results

### Demographic data

An average of 4.17 million datapoints (49.75% male) per year and a total of 41.66 million person years were available for analysis during the study period. At the start of participants’ eligibility, 19.4% of them were children aged 0–17 years. The median age of the study participants at the start of each year was 42 (Interquartile range:23–59) years.

Across the 10 years, on average a majority of the individuals (46.8%) self-identified as white. On average over the years, black, mixed race and ‘other’ minority groups constituted about 3% (1.5%, 1% and 0.50% respectively) and South Asians contributed to 2.38% of the data. Although ethnicity data were not available on nearly half (47.8%) of the registered individuals, an improvement in the recording of ethnicity was observed over the period of study: 53.9% of data on ethnicity was missing in 2010 compared to 46.1% missing in 2019).

Characteristics of the cross-sectional snapshot of patients at the start of each year between 2010 and 2019 are presented in additional data (S2).

### Prevalence of ARC, asthma and eczema

#### Prevalence amongst adults

**Fig. 1:**
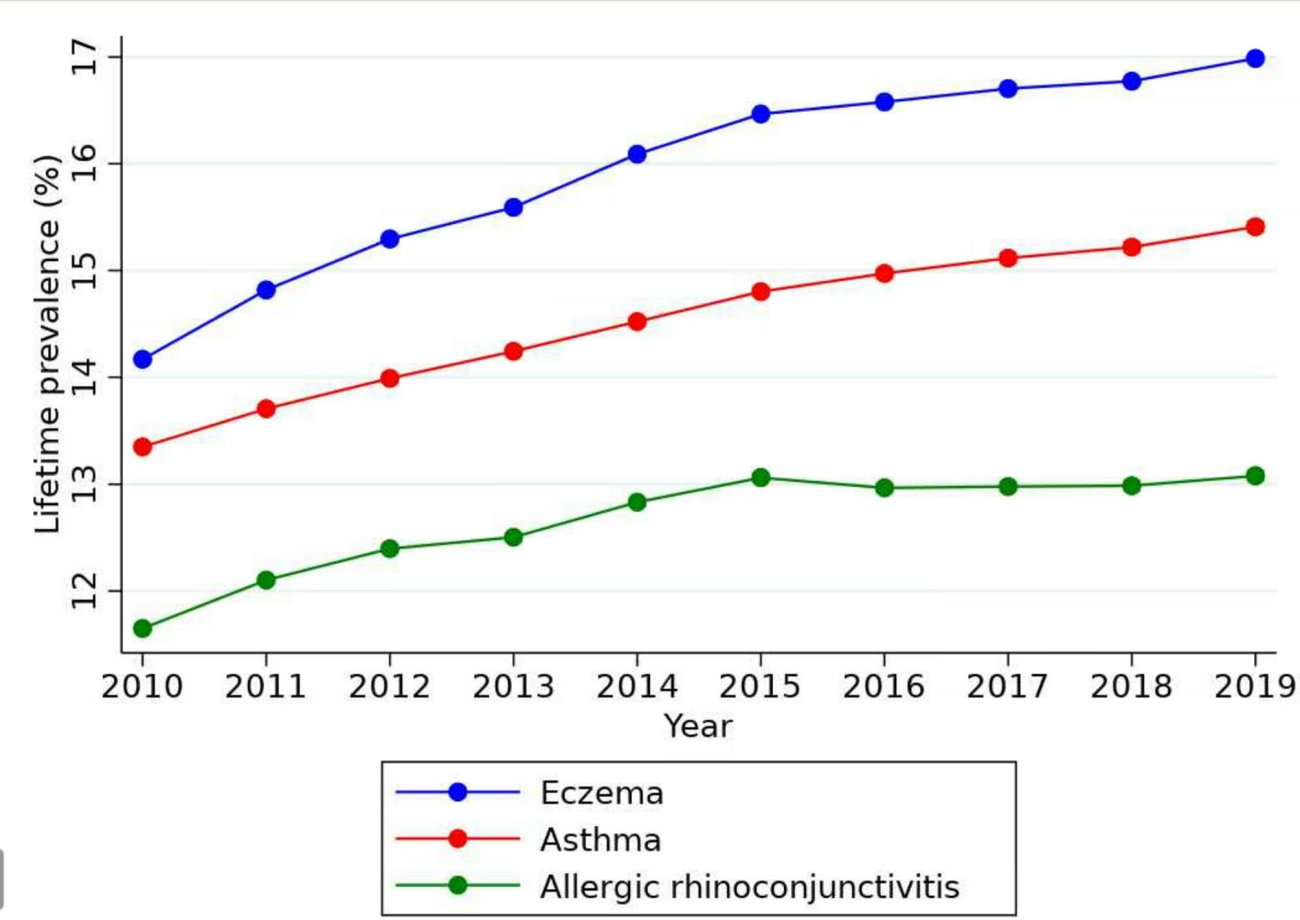
Trends in the prevalence of allergic rhinoconjunctivitis, asthma and eczema among adults (2010–2019)

Between the years 2010 and 2019, there was an increase in the prevalence of all three allergic conditions amongst the adult UK population (Fig. 1). The highest increase in prevalence was registered for eczema (18.32%) followed by asthma (14.02%) and ARC (11.48%).

By 2018 one in every 8 UK adults (12.99%) had ARC. The prevalence of asthma and eczema were higher, with 1 in every 7 (15.22%) and 1 in every 6 (16.77%) being diagnosed with these conditions respectively (Fig. 1; Table 1, additional data S3).

**Table 1:** Comparison of prevalence rates for adults between 2010–2018

	Prevalence * (in year 2010)	Prevalence * (in year 2018)	% change	Proportion with allergy in 2018
ARC	116.5	129.87	11.48	1 in 8
Asthma	133.48	152.19	14.02	1 in 7
Eczema	141.68	167.73	18.39	1 in 6

*prevalence per 1,000 persons

#### Prevalence amongst children

**Fig. 2:**
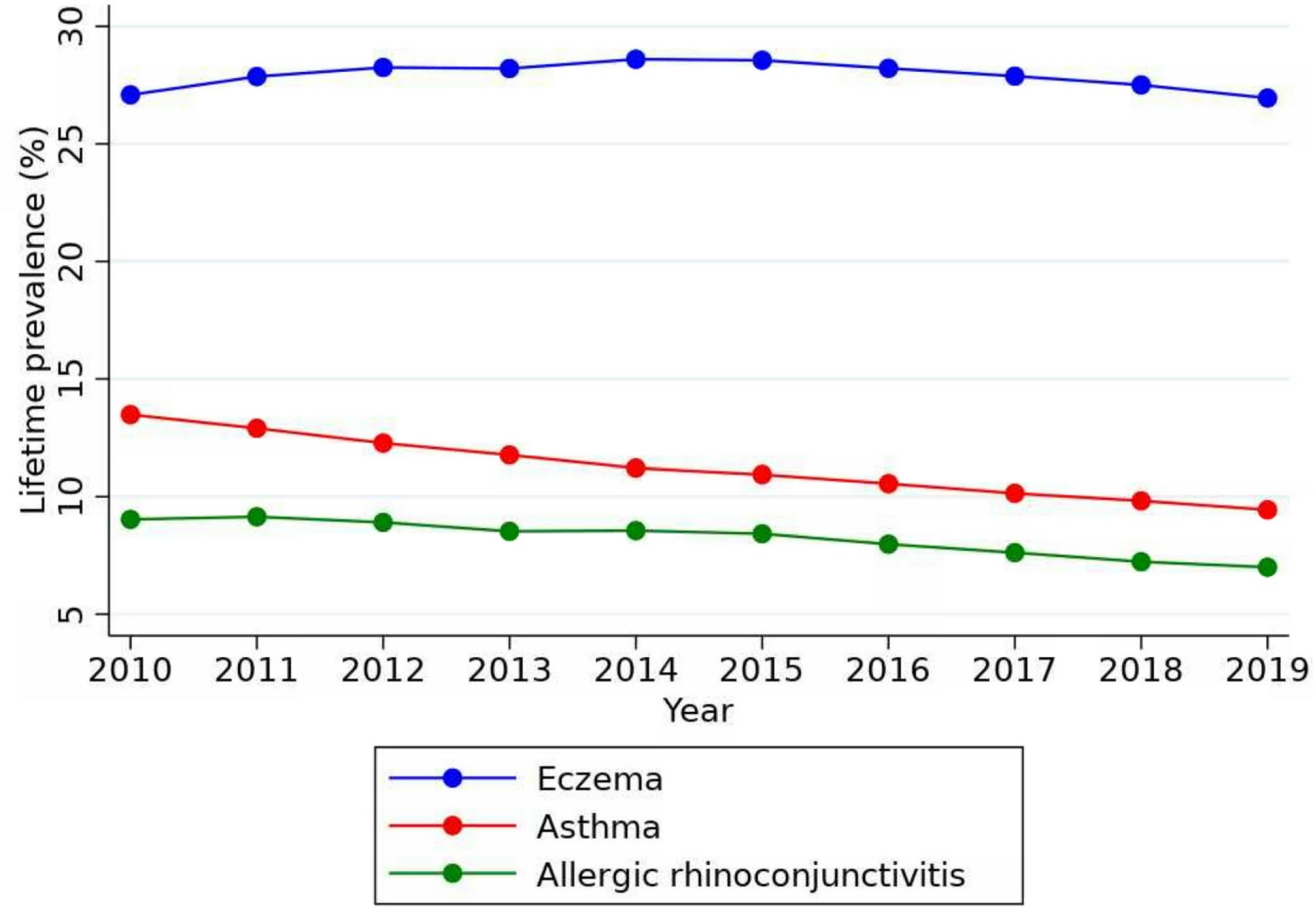
Trends in the prevalence of allergic rhinoconjunctivitis, asthma and eczema among children (2010–2019)

During the last decade, there has been a decrease in the prevalence of GP-diagnosed ARC and asthma amongst UK children (Fig. 2). The prevalence of eczema has increased slightly with one in 4 children (27.51%) diagnosed as having eczema in 2014. The prevalence of ARC and asthma was lower, with one in every 14 (7.23%) and 10 (9.82%) children being diagnosed with these conditions respectively. (Fig. 2; Table 2, additional data S4).

**Table 2:** Comparison of prevalence rates for children between 2010–2018

	Prevalence * (in year 2010)	Prevalence * (in year 2018)	% change	Proportion with allergy in 2018
ARC	90.34	72.33	-19.94	1 in 14
Asthma	134.78	98.24	-27.11	1 in 10
Eczema	270.86	275.1	1.57	1 in 4

*prevalence per 1,000 persons

### Risk factors associated with diagnosis of allergy among adults

In a cross-sectional snapshot of eligible adult patients at the start of 2018, the adjusted odds of being diagnosed with ARC were highest in adult patients between the ages of 30–50. In comparison to patients aged < 30 years, the adjusted odds ratios (OR) of patients aged 30–40, 40–50 and ≥ 50 years were 1.07 (95% CI 1.06–1.09), 1.06 (95% CI 1.05–1.07) and 0.75 (95% CI 0.75–0.76) respectively. Asthma and eczema, on the other hand, were more commonly diagnosed among young adults (aged < 30 years). Compared to men, women were at a 9%, 3% and 25% higher odds of being diagnosed with ARC, asthma and eczema [aOR: 1.09 (95% CI 1.08–1.10), 1.03 (1.02–1.04) and 1.25 (1.24–1.26)] respectively (Table 3).

**Table 3:** Risk factors for allergic rhiniconjuncitivits, asthma and eczema among adults

	ARC*	Asthma	Eczema
Age Category			
*< 30 years*	*Ref*	*Ref*	*Ref*
30–40 years	1.07 (1.06–1.09)	0.80 (0.79–0.81)	0.63 (0.62–0.63)
40–50 years	1.06 (1.05–1.07)	0.67 (0.66–0.68)	0.59 (0.59–0.60)
>=50 years	0.75 (0.75–0.76)	0.61 (0.60–0.61)	0.65 (0.64–0.66)
Gender			
*Men*	*Ref*	*Ref*	*Ref*
Women	1.09 (1.08–1.10)	1.03 (1.02–1.04)	1.25 (1.24–1.26)
Ethnicity			
*Caucasian*	*Ref*	*Ref*	*Ref*
Black Afro-caribbean	1.57 (1.53–1.62)	0.57 (0.55–0.59)	0.71 (0.69–0.74)
South Asian	1.49 (1.45–1.52)	0.67 (0.65–0.69)	1.08 (1.05–1.10)
Mixed Race	1.21 (1.17–1.26)	0.42 (0.40–0.44)	0.80 (0.77–0.83)
Chinese/middle eastern/others	1.31 (1.24–1.37)	0.82 (0.78–0.87)	0.93 (0.88–0.98)
Missing	1.07 (1.06–1.08)	0.95 (0.95–0.96)	1.09 (1.08–1.10)
Region			
*London*	*Ref*	*Ref*	*Ref*
East of England	1.21 (1.18–1.24)	1.25 (1.22–1.28)	1.24 (1.21–1.27)
Northwest of England	1.19 (1.16–1.21)	1.25 (1.23–1.28)	1.34 (1.31–1.36)
Northern Ireland	0.85 (0.84–0.87)	1.10 (1.08–1.12)	0.94 (0.93–0.96)
Scotland	0.90 (0.89–0.92)	1.15 (1.14–1.17)	1.01 (1.00-1.03)
South Central England	1.39 (1.36–1.43)	1.26 (1.23–1.29)	1.56 (1.53–1.60)
Southeast Coast of England	0.99 (0.98–1.01)	1.02 (1.00-1.03)	1.03 (1.01–1.04
Southwest of England	1.26 (1.23–1.29)	1.40 (1.37–1.43)	1.74 (1.70–1.78)
Wales	0.97 (0.96–0.99)	1.29 (1.27–1.31)	1.21 (1.19–1.23)
West Midlands	1.35 (1.33–1.38)	1.14 (1.11–1.16)	1.36 (1.34–1.39)
Yorkshire & Humber	0.55 (0.49–0.62)	1.28 (1.18–1.38)	0.63 (0.58–0.70)
Previous diagnosis of other allergic conditions			
ARC*	NA	2.68 (2.66–2.71)	1.96 (1.94–1.97)
Asthma	2.68 (2.66–2.71)	NA	1.68 (1.66–1.69)
Eczema	1.95 (1.94–1.97)	1.68 (1.66–1.69)	NA
Food allergy	1.78 (1.73–1.84)	2.40 (2.33–2.47)	1.93 (1.88–1.99)
Drug allergy	1.31 (1.30–1.32)	1.61 (1.59–1.62)	1.33 (1.32–1.34)
Urticaria	1.61 (1.58–1.63)	1.29 (1.28–1.31)	1.85 (1.82–1.87)
Anaphylaxis	1.28 (1.20–1.37)	1.76 (1.66–1.87)	1.18 (1.11–1.25)

*ARC = Allergic rhinoconjunctivitis

In comparison to Caucasians, adults belonging to ethnic minorities were at a higher risk of being diagnosed with ARC [aOR for Black Afro-Caribbean: 1.57 (95% CI 1.53–1.62), South Asian: 1.49 (95% CI 1.45–1.52), mixed race: 1.21 (95% CI 1.17–1.26), other ethnic minority: 1.31 (95% CI 1.24–1.37)], while at a lower risk of being diagnosed with asthma [aOR for Black Afro-Caribbean: 0.57 (95% CI 0.55–0.59), South Asian: 0.67 (95% CI 0.65–0.69), mixed race: 0.42 (95% CI 0.40–0.44), other ethnic minority: 0.82 (95% CI 0.78–0.87)] and eczema [aOR for Black Afro-Caribbean: 0.71 (95% CI 0.69–0.74), mixed ethnicity: 0.80 (95% CI 0.77–0.83), other ethnic minority: 0.93 (95% CI 0.88–0.98)](Table 3).

Adult patients from other regions such as Yorkshire and Humber were less likely to be diagnosed with ARC and eczema compared to patients from London [aOR: 0.55 (0.49–0.62) and 0.63 (0.58–0.70) respectively], while those from London were less likely to be diagnosed with asthma. Diagnosis of any of the allergic conditions discussed (i.e., ARC, eczema and asthma) significantly increased the odds amongst adults, as well as children, of being diagnosed with either one of these allergies (ARC, eczema or asthma) or another allergy / related condition, such as food allergy, anaphylaxis, drug allergies or urticaria (Table 3).

### Risk factors associated with diagnosis of allergy among children

The adjusted odds of being diagnosed with ARC, asthma and eczema were higher among children aged 10–18 years old compared to < 10 year olds [aOR: 3.23 (95% CI 3.16–3.31), 2.56 (95% CI 2.51–2.61) and 1.08 (95% CI 1.07–1.09) respectively], and lower in girls compared to boys [aOR: 0.73 (95% CI 0.71–0.74), 0.75 (95 CI 0.73–0.76) and 0.95 (95% CI 0.94–0.96) respectively] (Table 4).

**Table 4:** Risk factors for allergic rhiniconjuncitivits, asthma and eczema among children

	ARC*	Asthma	Eczema
Age Category			
*< 10 years*	*Ref*	*Ref*	*Ref*
10–18 years	3.23 (3.16–3.31)	2.56 (2.51–2.61)	1.08 (1.07–1.09)
Sex			
*Boys*	*Ref*	*Ref*	*Ref*
Girls	0.73 (0.71–0.74)	0.75 (0.73–0.76)	0.95 (0.94–0.96)
Ethnicity			
*Caucasian*	*Ref*	*Ref*	*Ref*
Black Afro-caribbean	2.25 (2.12–2.38)	0.77 (0.72–0.82)	1.30 (1.25–1.35)
South Asian	1.90 (1.80-2.00)	1.01 (0.96–1.06)	1.22 (1.18–1.27)
Mixed Race	1.56 (1.42–1.70)	0.64 (0.58–0.71)	1.21 (1.14–1.28)
Chinese/middle eastern/others	1.34 (1.24–1.46)	1.06 (0.99–1.15)	1.07 (1.02–1.13)
Missing	1.08 (1.05–1.10)	0.89 (0.87–0.90)	1.04 (1.03–1.06)
Region			
East of England	1.13 (1.06–1.20)	1.41 (1.34–1.49)	1.23 (1.19–1.27)
London	Ref	Ref	Ref
Northwest of England	1.00 (0.95–1.06)	1.44 (1.37–1.51)	1.21 (1.17–1.25)
Northern Ireland	0.95 (0.90–0.99)	1.73 (1.66–1.80)	0.78 (0.76–0.81)
Scotland	0.75 (0.73–0.78)	1.37 (1.33–1.42)	0.77 (0.75–0.79)
South Central England	1.28 (1.21–1.35)	1.32 (1.25–1.40)	1.35 (1.31–1.40)
Southeast Coast of England	1.01 (0.97–1.05)	1.15 (1.10–1.20)	1.07 (1.04–1.09)
Southwest of England	0.98 (0.92–1.05)	1.47 (1.39–1.56)	1.29 (1.24–1.34)
Wales	1.06 (1.02–1.10)	1.39 (1.34–1.44)	1.10 (1.08–1.12)
West Midlands	1.41 (1.35–1.48)	1.27 (1.21–1.33)	1.19 (1.15–1.22)
Yorkshire & Humber	0.15 (0.08–0.29)	1.29 (1.01–1.64)	0.24 (0.19–0.31)
Previous diagnosis of other allergic conditions			
ARC*	NA	2.66 (2.59–2.73)	2.07 (2.03–2.12)
Asthma	2.66 (2.60–2.73)	NA	1.83 (1.80–1.87)
Eczema	2.10 (2.06–2.14)	1.85 (1.81–1.88)	NA
Food allergy	1.87 (1.78–1.97)	2.85 (2.73–2.97)	3.74 (3.60–3.88)
Drug allergy	1.40 (1.35–1.46)	1.71 (1.65–1.77)	1.36 (1.33–1.40)
Urticaria	1.56 (1.50–1.62)	1.30 (1.26–1.35)	1.53 (1.49–1.57)
Anaphylaxis	1.32 (1.09–1.60)	1.87 (1.57–2.22)	1.54 (1.30–1.82)

*ARC = Allergic rhinoconjuctivitis

Similar to adults, children from ethnic minority groups were at higher odds of being diagnosed with ARC, and at lower odds of being diagnosed with asthma. However, unlike adults, children from ethnic minority groups were at higher odds of being diagnosed with eczema in comparison with their respective Caucasian counterparts [aOR for Black Afro-Caribbean: 1.30 (95% CI 1.25–1.35), South Asian: 1.22 (95% CI 1.18–1.27), mixed ethnicity: 1.21 (95 CI 1.14–1.28) and other ethnicity: 1.07 (95 CI 1.02–1.13)] (Table 4).

Odds of developing any of the three allergic conditions across the various regions of the UK were similar between adults and children. Children with previous diagnosis of any other allergy were at higher odds of being diagnosed with ARC, asthma and eczema.

## Discussion

Our analysis shows that a substantial proportion of the UK population is now affected by one of either ARC, eczema or asthma, and that the allergy burden on the NHS in the UK is increasing.

The prevalence of eczema amongst children remains quite high with a quarter of all UK children having been diagnosed with this condition. The burden of childhood asthma and ARC is also substantial. However, there is a variation in allergy prevalence between different age groups. The regression analysis performed for the year 2018 showed that ARC is more commonly diagnosed in older children (aged 10-18yrs) compared to younger (< 10yrs old) children. Ethnicity also appears to be a significant variable, with Caucasians less likely to be diagnosed with ARC and more likely to have asthma compared with other ethnic groups. Caucasian adults also appear to have a lower prevalence of eczema. There were regional variations in allergies with asthma and eczema being more common in most other UK regions than in London. The prevalence of ARC was highest in the West Midlands and South-Central England.

Previous work has shown that individuals who suffer from one of these allergic conditions are more likely to suffer from an additional allergy [29]. An Irish study used the standardized ISAAC questionnaire to conclude that the co-existence of atopic conditions amongst children aged 6–7 years had increased between 2002 and 2007 [30]. The increased risk of developing food allergy in individuals diagnosed with other allergies has previously been described [31, 32]. Our study showed that allergies tend to cluster in individuals and that there was a higher chance of being diagnosed with other allergies/ atopic conditions (including food allergy, drug allergy, urticaria) in individuals with either ARC, eczema or asthma, across all age groups.

There is a substantial economic impact of these conditions on health care systems and a considerable personal burden and impact on the quality of life of affected individuals [33–37].  A survey of adult patients with allergic rhinitis in 5 European countries (including the UK) showed that a majority of them reported tiredness, irritability, anxiety and depression [38]. Many also reported sleep disruption and a detrimental impact on school/ work performance [38]. Other studies have confirmed a reduction in quality of life due to ARC [39, 40] asthma [41, 42] and eczema.

The increase in co-morbidity of multiple allergies has implications for clinical services also. Individuals with multiple and complicated allergies have higher morbidity and are more likely to benefit from allergy specialist services [43, 44]. However, as has been discussed in previous publications [45, 46] there are significant issues with specialist availability for paediatric and adult allergy services in the UK. Moreover, there are very few centres in the UK that offer combined clinics with allergy/ ENT/ respiratory specialists.

The International Study of Asthma and Allergies in Childhood (ISAAC), a large multi-centre study estimating the prevalence of self-reported allergic conditions, found that whilst eczema and asthma prevalence had levelled off or decreased in areas with previous high prevalence (such as in the UK) [47] the prevalence of allergic rhinitis– especially in younger children- has increased in most countries [48]. A previous analysis of primary care databases [22] has shown a possible stabilisation in the prevalence of allergic rhinitis and eczema in the UK. Estimates, therefore, appear to vary significantly based on the method used (e.g. self-reporting versus clinical diagnosis), definition of allergy itself (blood test positivity versus self-reporting of symptoms by patient versus challenge test in a clinic), age groups studied, or, in the case of retrospective cohort studies (similar to those presented in this study), source and setting of data [49]. The reason for the steadily reducing prevalence of ARC amongst children during the study period is not clear. The recent challenges difficulty in access to UK primary care could discourage parents from contacting their general practitioners for relatively minor ailments [50] however this is not likely to provide a full explanation for this observation.

A previous publication using UK primary care databases (GPRD) [51] has shown high incidence density of ARC, asthma and eczema in a cohort of children. Although this work did not explore risk factors or explore prevalence trends in the diagnosis of these conditions, it did demonstrate a growing problem of multiple allergies amongst children.

A major strength of our study is the use of a large, representative national primary care database (THIN) which includes data from across the UK. The dataset provided longitudinal data on over 4.17 million individuals per year, allowing estimations with high statistical power. THIN provides diagnoses documented by a qualified clinician and could be considered to provide a more credible estimate than that provided by self-reported allergies. However, many mild allergies are self-managed by patients (and parents) at home using over the counter medication without ever consulting general practitioners. Thus, the estimates in this study may not accurately reflect the burden of disease in the community. Also, these data reflect diagnoses made in primary care and may reflect an over or under reporting of allergies. Moreover, as with any health database, there could be issues with accuracy of coding and missing data [52].

THIN is a dynamic database and various GP practices may have joined or left the dataset during the study period. Over the last decade, there has been a gradual decrease in the total number of GP practices (particularly those in some regions of England) subscribing to THIN and this is reflected in a reduction in the overall numbers of individuals within the dataset (see additional data S2). This may have affected the estimates provided, particularly regional estimates. Confounders such as family history, smoking status, area of residence (urban or rural) could be important but were not considered in this analysis. Also, ethnicity data was not specified for about 50% of those included in the dataset. It should be noted, however, that data from THIN has been shown consistently to be of high quality and completeness as well as generalisable to the UK population [25, 53–55].

## Conclusions

Our study shows that the burden of ARC, asthma and eczema in the UK is substantial. There are age, gender, ethnicity and region-based differences in the prevalence of allergies in the UK. Individuals diagnosed with these conditions are at higher risk of developing other allergies. There is growing number of individuals with multiple and complex allergies who would benefit from specialist input. These data support the call for urgent improvement to allergy services and pathways in the UK.

## Electronic supplementary material

Below is the link to the electronic supplementary material.

Supplementary Material 1

## Data Availability

No datasets were generated or analysed during the current study.
