# Supplementary material for: Prevalence trends and risk factors for allergic rhinoconjunctivitis, asthma and eczema in the UK
Source: Allergy Asthma Clin Immunol. 2025 Jul 7;21:31. doi: 10.1186/s13223-025-00975-2 (PMC12232741; doi:10.1186/s13223-025-00975-2)
Supplement: Supplementary file 1 — Supplementary Material 1 [file 13223_2025_975_MOESM1_ESM.docx]

Supplementary material:

Supplement S1

**Allergic Rhinoconjunctivitis**

| Code | Description |
| --- | --- |
| F4C1300 | Vernal conjunctivitis |
| H171.16 | House dust mite allergy |
| F4C0611 | Acute allergic conjunctivitis |
| SN5A.00 | Oral allergy syndrome |
| F4C1400 | Other chronic allergic conjunctivitis |
| H171.12 | Dander (animal) allergy |
| H171.13 | Feather allergy |
| H171.14 | Hay fever - other allergen |
| H171.15 | House dust allergy |
| H17..00 | Allergic rhinitis |
| H172.00 | Allergic rhinitis due to unspecified allergen |
| H170.00 | Allergic rhinitis due to pollens |
| H171.11 | Cat allergy |
| Hyu2100 | [X]Other allergic rhinitis |
| Hyu2000 | [X]Other seasonal allergic rhinitis |
| H330.14 | Pollen asthma |
| F4C0600 | Acute atopic conjunctivitis |
| F4A3100 | Vernal conjunctivitis of limbus and cornea |
| F4C1411 | Allergic conjunctivitis |
| H171000 | Allergy to animal |
| H171100 | Dog allergy |
| H17..11 | Perennial rhinitis |
| H17..12 | Allergic rhinosinusitis |
| H172.11 | Hay fever - unspecified allergen |
| H170.12 | Pollinosis |
| H170.11 | Hay fever - pollens |
| H171.00 | Allergic rhinitis due to other allergens |
| H17z.00 | Allergic rhinitis NOS |

**Asthma**

| Code | Description |
| --- | --- |
| 8795.00 | Asthma control step 2 |
| H330000 | Extrinsic asthma without status asthmaticus |
| H331.11 | Late onset asthma |
| 679J.00 | Health education - asthma |
| H334.00 | Brittle asthma |
| 663d.00 | Emergency asthma admission since last appointment |
| 9OJ5.00 | Asthma monitor 2nd letter |
| 8B3j.00 | Asthma medication review |
| H33z100 | Asthma attack |
| 1782.00 | Asthma trigger - tobacco smoke |
| 66YQ.00 | Asthma monitoring by nurse |
| 663t.00 | Asthma causes daytime symptoms 1 to 2 times per month |
| 388t.00 | Royal College of Physicians asthma assessment |
| 663Q.00 | Asthma not limiting activities |
| 66YC.00 | Absent from work or school due to asthma |
| 663V200 | Moderate asthma |
| 66YK.00 | Asthma follow-up |
| H330111 | Extrinsic asthma with asthma attack |
| H33..11 | Bronchial asthma |
| 663V.00 | Asthma severity |
| H33zz00 | Asthma NOS |
| 663q.00 | Asthma daytime symptoms |
| H331.00 | Intrinsic asthma |
| 9OJ2.00 | Refuses asthma monitoring |
| H33z111 | Asthma attack NOS |
| 1785.00 | Asthma trigger - damp |
| 663y.00 | Number of asthma exacerbations in past year |
| 663N.00 | Asthma disturbing sleep |
| 663P100 | Asthma limits activities 1 to 2 times per week |
| 663N200 | Asthma disturbs sleep frequently |
| 663j.00 | Asthma - currently active |
| 9OJB.00 | Asthma monitorng invit SMS (short message servce) txt messge |
| 8797.00 | Asthma control step 4 |
| H331111 | Intrinsic asthma with asthma attack |
| 1780.00 | Aspirin induced asthma |
| 663W.00 | Asthma prophylactic medication used |
| 663..11 | Asthma monitoring |
| H332.00 | Mixed asthma |
| 679J100 | Health education - structured asthma discussion |
| 173c.00 | Occupational asthma |
| H33z.00 | Asthma unspecified |
| 1788 | Asthma trigger - cold air |
| 9OJ3.00 | Asthma monitor offer default |
| 663r.00 | Asthma causes night symptoms 1 to 2 times per month |
| 661N100 | Asthma self-management plan review |
| 66Y9.00 | Step up change in asthma management plan |
| 663O.00 | Asthma not disturbing sleep |
| 66Yz000 | Asthma management plan declined |
| 66YA.00 | Step down change in asthma management plan |
| 9NNX.00 | Under care of asthma specialist nurse |
| 663V100 | Mild asthma |
| H331100 | Intrinsic asthma with status asthmaticus |
| H330011 | Hay fever with asthma |
| 66Yq.00 | Asthma causes night time symptoms 1 to 2 times per week |
| 8794.00 | Asthma control step 1 |
| H33z200 | Late-onset asthma |
| 679J200 | Health education - structured patient focused asthma discuss |
| 663w.00 | Asthma limits walking up hills or stairs |
| H33z.11 | Hyperreactive airways disease |
| 9OJ8.00 | Asthma monitor phone invite |
| 1783.00 | Asthma trigger - warm air |
| 663P000 | Asthma limits activities 1 to 2 times per month |
| H331z00 | Intrinsic asthma NOS |
| 663N100 | Asthma disturbs sleep weekly |
| 663U.00 | Asthma management plan given |
| 178A.00 | Asthma trigger - airborne dust |
| 9OJA.11 | Asthma monitored |
| 66YJ.00 | Asthma annual review |
| 178..00 | Asthma trigger |
| 8791.00 | Further asthma - drug prevent. |
| 663p.00 | Asthma treatment compliance unsatisfactory |
| 9OJ1.00 | Attends asthma monitoring |
| 38DT.00 | Asthma control questionnaire |
| H330.00 | Extrinsic (atopic) asthma |
| 9OJ..11 | Asthma clinic administration |
| 679J000 | Health education - asthma self management |
| 1786.00 | Asthma trigger - animals |
| 663x.00 | Asthma limits walking on the flat |
| 66Yp.00 | Asthma review using Roy Colleg of Physicians three questions |
| 388t000 | Royal College Physician asthma assessment 3 question score |
| 8H2P.00 | Emergency admission, asthma |
| 663V000 | Occasional asthma |
| 8796.00 | Asthma control step 3 |
| 663m.00 | Asthma accident and emergency attendance since last visit |
| 66Ys.00 | Asthma never causes night symptoms |
| 9OJZ.00 | Asthma monitoring admin.NOS |
| H331000 | Intrinsic asthma without status asthmaticus |
| H335.00 | Chronic asthma with fixed airflow obstruction |
| 9OJ..00 | Asthma monitoring admin. |
| 38DL.00 | Asthma control test |
| 663e.00 | Asthma restricts exercise |
| 9OJ6.00 | Asthma monitor 3rd letter |
| 66YP.00 | Asthma night-time symptoms |
| 663u.00 | Asthma causes daytime symptoms 1 to 2 times per week |
| 1781.00 | Asthma trigger - pollen |
| 1789.00 | Asthma trigger - respiratory infection |
| 663N000 | Asthma causing night waking |
| 173A.00 | Exercise induced asthma |
| 1O2..00 | Asthma confirmed |
| H330100 | Extrinsic asthma with status asthmaticus |
| 663n.00 | Asthma treatment compliance satisfactory |
| H33..00 | Asthma |
| 38DV.00 | Mini asthma quality of life questionnaire |
| H33zz13 | Allergic bronchitis NEC |
| 66Yr.00 | Asthma causes symptoms most nights |
| H33zz12 | Allergic asthma NEC |
| H33zz11 | Exercise induced asthma |
| 66Y5.00 | Change in asthma management plan |
| 663f.00 | Asthma never restricts exercise |
| 38QM.00 | Childhood Asthma Control Test |
| H33z000 | Status asthmaticus NOS |
| 663v.00 | Asthma causes daytime symptoms most days |
| 1784.00 | Asthma trigger - emotion |
| 9OJ7.00 | Asthma monitor verbal invite |
| H47y000 | Detergent asthma |
| H330z00 | Extrinsic asthma NOS |
| 663O000 | Asthma never disturbs sleep |
| 66YE.00 | Asthma monitoring due |
| 66Yu.00 | Number days absent from school due to asthma in past 6 month |
| 178B.00 | Asthma trigger - exercise |
| H35y700 | Wood asthma |
| 9OJA.00 | Asthma monitoring check done |
| H330.14 | Pollen asthma |
| 8798.00 | Asthma control step 5 |
| H330.13 | Hay fever with asthma |
| 66YZ.00 | Does not have asthma management plan |
| H330.12 | Childhood asthma |
| H330.11 | Allergic asthma |
| H333.00 | Acute exacerbation of asthma |
| 8CR0.00 | Asthma clinical management plan |
| H33z011 | Severe asthma attack |
| 1787.00 | Asthma trigger - seasonal |
| 9OJ4.00 | Asthma monitor 1st letter |
| H312000 | Chronic asthmatic bronchitis |
| 663s.00 | Asthma never causes daytime symptoms |
| 66YR.00 | Asthma monitoring by doctor |
| 663P.00 | Asthma limiting activities |
| 661M100 | Asthma self-management plan agreed |
| 663P200 | Asthma limits activities most days |
| 663V300 | Severe asthma |
| 8CMA000 | Patient has a written asthma personal action plan |
| 173d.00 | Work aggravated asthma |

**Eczema**

| Code | Description |
| --- | --- |
| F502400 | Acute eczematoid otitis extern |
| M11..00 | Atopic dermatitis and related conditions |
| M112.00 | Infantile eczema |
| M114.00 | Allergic (intrinsic) eczema |
| M102.11 | Pustular eczema |
| M12z111 | Discoid eczema |
| F4D3112 | Contact eczema - eyelids |
| M11A.00 | Asteatotic eczema |
| 26C4.00 | Nipple eczema |
| M12..00 | Contact dermatitis and other eczemas |
| F502411 | Eczema of external ear |
| M11z.00 | Atopic dermatitis NOS |
| Myu2.00 | [X]Dermatitis and eczema |
| M119.00 | Discoid eczema |
| Myu2200 | [X]Exacerbation of eczema |
| M111.00 | Atopic dermatitis/eczema |
| M117.00 | Neurodermatitis - atopic |
| M07y.11 | Pustular eczema |
| M115.00 | Besnier's prurigo |
| M113.00 | Flexural eczema |
| M12z200 | Infected eczema |
| M12z300 | Hand eczema |
| M12z100 | Eczema NOS |
| F4D3000 | Eczematous eyelid dermatitis |
| M12..12 | Contact eczema |
| M12z400 | Erythrodermic eczema |

Supplement S2

**Characteristics of the cross-sectional cohort at the start of each year between 2010 and 2019***

| **Year** | **2010** | **2011** | **2012** | **2013** | **2014** | **2015** | **2016** | **2017** | **2018** | **2019** |
| --- | --- | --- | --- | --- | --- | --- | --- | --- | --- | --- |
| **Eligible patients (n)** | **5201318** | **5189619** | **5300984** | **5428998** | **5125491** | **4807142** | **4044215** | **3669603** | **3262100** | **3073502** |
| **Age [Mean(SD)]** | 40.72 (22.97) | 40.76 (23.05) | 40.76 (23.13) | 40.75 (23.16) | 40.89 (23.24) | 40.93 (23.30) | 41.00 (23.30) | 40.88 (23.27) | 40.97 (23.25) | 41.17 (23.30) |
| **Age [Median(IQR)]** | 40.00 (22.00-58.00) | 40.00 (22.00-58.00) | 40.00 (22.00-58.00) | 40.00 (22.00-58.00) | 41.00 (22.00-58.00) | 41.00 (22.00-58.00) | 41.00 (22.00-58.00) | 40.00 (22.00-58.00) | 40.00 (22.00-58.00) | 41.00 (22.00-59.00) |
| **Age categories** |  |  |  |  |  |  |  |  |  |  |
| **Children (<18 yrs)** | 1038186 (19.96) | 1041219 (20.06) | 1071088 (20.21) | 1101718 (20.29) | 1042723 (20.34) | 983026 (20.45) | 826986 (20.45) | 755974 (20.60) | 669137 (20.51) | 627612 (20.42) |
| **Adults (>= 18 yrs)** | 4163132 (80.04) | 4148400 (79.94) | 4229896 (79.79) | 4327280 (79.71) | 4082768 (79.66) | 3824116 (79.55) | 3217229 (79.55) | 2913629 (79.40) | 2592963 (79.49) | 2445890 (79.58) |
| **Sex** |  |  |  |  |  |  |  |  |  |  |
| **Men, n(%)** | 2589182 (49.78) | 2579205 (49.70) | 2629132 (49.60) | 2692129 (49.59) | 2538912 (49.54) | 2380360 (49.52) | 2003734 (49.55) | 1819141 (49.57) | 1618957 (49.63) | 1524472 (49.60) |
| **Women, n(%)** | 2612136 (50.22) | 2610414 (50.30) | 2671852 (50.40) | 2736869 (50.41) | 2586579 (50.46) | 2426782 (50.48) | 2040481 (50.45) | 1850462 (50.43) | 1643143 (50.37) | 1549030 (50.40) |
| **Ethnicity** |  |  |  |  |  |  |  |  |  |  |
| **White** | 2268617 (43.62) | 2373906 (45.74) | 2490455 (46.98) | 2630686 (48.46) | 2469215 (48.18) | 2329425 (48.46) | 1986390 (49.12) | 1810718 (49.34) | 1587918 (48.68) | 1507004 (49.03) |
| **Black Afro-Caribbean** | 76749 (1.48) | 83055 (1.60) | 88382 (1.67) | 92065 (1.70) | 85203 (1.66) | 77480 (1.61) | 63789 (1.58) | 62030 (1.69) | 54541 (1.67) | 48224 (1.57) |
| **South Asian** | 120871 (2.32) | 133249 (2.57) | 144747 (2.73) | 151009 (2.78) | 140223 (2.74) | 126255 (2.63) | 98266 (2.43) | 91326 (2.49) | 80457 (2.47) | 70683 (2.30) |
| **Mixed Race** | 48470 (0.93) | 53427 (1.03) | 58854 (1.11) | 63120 (1.16) | 59769 (1.17) | 53204 (1.11) | 43702 (1.08) | 41634 (1.13) | 39321 (1.21) | 37216 (1.21) |
| **Other minorities** | 26746 (0.51) | 29658 (0.57) | 32990 (0.62) | 35893 (0.66) | 34558 (0.67) | 31591 (0.66) | 26631 (0.66) | 25886 (0.71) | 22964 (0.70) | 21066 (0.69) |
| **Missing** | 2659865 (51.14) | 2516324 (48.49) | 2485556 (46.89) | 2456225 (45.24) | 2336523 (45.59) | 2189187 (45.54) | 1825437 (45.14) | 1638009 (44.64) | 1476899 (45.27) | 1389309 (45.20) |
| **General Practice Region** |  |  |  |  |  |  |  |  |  |  |
| **East Midlands** | 160910 (3.09) | 102729 (1.98) | 97536 (1.84) | 73407 (1.35) | 11882 (0.23) | 0 (0%) | 0 (0%) | 0 (0%) | 0 (0%) | 0 (0%) |
| **East of England** | 316885 (6.09) | 316229 (6.09) | 301861 (5.69) | 283502 (5.22) | 243401 (4.75) | 217719 (4.53) | 116199 (2.87) | 118790 (3.24) | 95035 (2.91) | 58116 (1.89) |
| **London** | 663317 (12.75) | 685302 (13.21) | 692076 (13.06) | 704578 (12.98) | 679804 (13.26) | 555738 (11.56) | 429355 (10.62) | 408350 (11.13) | 359423 (11.02) | 295402 (9.61) |
| **North East** | 106960 (2.06) | 104231 (2.01) | 104926 (1.98) | 90062 (1.66) | 73161 (1.43) | 60856 (1.27) | 38876 (0.96) | 39305 (1.07) | 0 (0%) | 0 (0%) |
| **North West** | 503171 (9.67) | 493111 (9.50) | 479055 (9.04) | 482457 (8.89) | 469776 (9.17) | 411427 (8.56) | 296818 (7.34) | 214964 (5.86) | 165070 (5.06) | 134491 (4.38) |
| **Northern Ireland** | 209556 (4.03) | 214418 (4.13) | 216666 (4.09) | 230061 (4.24) | 232068 (4.53) | 256789 (5.34) | 260263 (6.44) | 263652 (7.18) | 266411 (8.17) | 269227 (8.76) |
| **Scotland** | 632426 (12.16) | 654759 (12.62) | 763080 (14.40) | 958841 (17.66) | 970432 (18.93) | 975598 (20.29) | 985538 (24.37) | 984345 (26.82) | 943560 (28.92) | 950864 (30.94) |
| **South Central** | 563094 (10.83) | 566718 (10.92) | 575634 (10.86) | 544637 (10.03) | 531215 (10.36) | 477317 (9.93) | 270649 (6.69) | 129900 (3.54) | 103868 (3.18) | 73129 (2.38) |
| **South East Coast** | 546258 (10.50) | 546311 (10.53) | 549237 (10.36) | 544435 (10.03) | 533360 (10.41) | 513332 (10.68) | 507488 (12.55) | 428127 (11.67) | 334862 (10.27) | 329426 (10.72) |
| **South West** | 405571 (7.80) | 389362 (7.50) | 390032 (7.36) | 385529 (7.10) | 308231 (6.01) | 240425 (5.00) | 160521 (3.97) | 122379 (3.33) | 84266 (2.58) | 64272 (2.09) |
| **Wales** | 542673 (10.43) | 565559 (10.90) | 592643 (11.18) | 598660 (11.03) | 602555 (11.76) | 682810 (14.20) | 704030 (17.41) | 717500 (19.55) | 725556 (22.24) | 728650 (23.71) |
| **West Midlands** | 455529 (8.76) | 459788 (8.86) | 475209 (8.96) | 481558 (8.87) | 422339 (8.24) | 367117 (7.64) | 236288 (5.84) | 220026 (6.00) | 178072 (5.46) | 163824 (5.33) |
| **Yorkshire & Humber** | 94968 (1.83) | 91102 (1.76) | 63029 (1.19) | 51271 (0.94) | 47267 (0.92) | 48014 (1.00) | 38190 (0.94) | 22265 (0.61) | 5977 (0.18) | 6101 (0.20) |

Supplement S3: Trends in prevalence of allergic rhinoconjunctivitis, asthma and eczema among adults

| **Year** | **Population (Denominator)** | **Allergic rhinoconjunctivitis** | | **Eczema** | | **Asthma** | |
| --- | --- | --- | --- | --- | --- | --- | --- |
|  |  | **Numerator** | **Prevalence per 1,000 patients  (95% CI)** | **Numerator** | **Prevalence per 1,000 patients  (95% CI)** | **Numerator** | **Prevalence per 1,000 patients  (95% CI)** |
| 2010 | 3896948 | 453980 | 116.5 (116.18-116.81) | 552133 | 141.68 (141.34-142.03 | 520145 | 133.48 (133.14-133.81 |
| 2011 | 3880055 | 469593 | 121.03 (120.7-121.35 | 574947 | 148.18 (147.83-148.53 | 531751 | 137.05 (136.71-137.39 |
| 2012 | 3947698 | 489392 | 123.97 (123.64-124.29 | 603810 | 152.95 (152.6-153.31 | 552271 | 139.9 (139.55-140.24 |
| 2013 | 4039948 | 505164 | 125.04 (124.72-125.36 | 629894 | 155.92 (155.56-156.27 | 575387 | 142.42 (142.08-142.77 |
| 2014 | 3808752 | 488757 | 128.32 (127.99-128.66 | 612839 | 160.9 (160.53-161.27 | 553073 | 145.21 (144.86-145.56 |
| 2015 | 3562699 | 465345 | 130.62 (130.27-130.97 | 586647 | 164.66 (164.28-165.05 | 527361 | 148.02 (147.65-148.39 |
| 2016 | 3006384 | 389819 | 129.66 (129.28-130.04 | 498433 | 165.79 (165.37-166.21 | 450119 | 149.72 (149.32-150.12 |
| 2017 | 2718773 | 352867 | 129.79 (129.39-130.19 | 454145 | 167.04 (166.6-167.48 | 410976 | 151.16 (150.74-151.59 |
| 2018 | 2428076 | 315328 | 129.87 (129.44-130.29 | 407270 | 167.73 (167.26-168.2 | 369535 | 152.19 (151.74-152.64 |
| 2019 | 2294050 | 300033 | 130.79 (130.35-131.22 | 389688 | 169.87 (169.38-170.35 | 353497 | 154.09 (153.63-154.56 |

Supplement S4: Trends in prevalence of allergic rhinoconjunctivitis, asthma and eczema among children

| **Year** | **Population (Denominator)** | **Allergic rhinoconjunctivitis** | | **Eczema** | | **Asthma** | |
| --- | --- | --- | --- | --- | --- | --- | --- |
|  |  | **Numerator** | **Prevalence per 1,000 patients  (95% CI)** | **Numerator** | **Prevalence per 1,000 patients  (95% CI)** | **Numerator** | **Prevalence per 1,000 patients  (95% CI)** |
| 2010 | 915019 | 82681 | 90.36 (89.77-90.95) | 247842 | 270.86 (269.95-271.77 | 123327 | 134.78 (134.08-135.48) |
| 2011 | 914992 | 83683 | 91.46 (90.87-92.05) | 254956 | 278.64 (277.72-279.56 | 118006 | 128.97 (128.28-129.66) |
| 2012 | 940228 | 83750 | 89.07 (88.50-89.65) | 265624 | 282.51 (281.6-283.42 | 115352 | 122.69 (122.02-123.35) |
| 2013 | 968859 | 82610 | 85.27 (84.71-85.82) | 273292 | 282.08 (281.18-282.97 | 113986 | 117.65 (117.01-118.29) |
| 2014 | 918337 | 78580 | 85.57 (85.00-86.14) | 262663 | 286.02 (285.1-286.94 | 102973 | 112.13 (111.48-112.78) |
| 2015 | 865720 | 72932 | 84.24 (83.66-84.83) | 247222 | 285.57 (284.62-286.52 | 94665 | 109.35 (108.69-110.01) |
| 2016 | 731837 | 58392 | 79.79 (79.17-80.41) | 206482 | 282.14 (281.11-283.17 | 77240 | 105.54 (104.84-106.25) |
| 2017 | 668910 | 50975 | 76.21 (75.57-76.84) | 186506 | 278.82 (277.75-279.9 | 67831 | 101.41 (100.68-102.13) |
| 2018 | 595884 | 43099 | 72.33 (71.67-72.99) | 163914 | 275.08 (273.94-276.21 | 58541 | 98.24 (97.49-99.00) |
| 2019 | 561190 | 39287 | 70.01 (69.34-70.67) | 151237 | 269.49 (268.33-270.65 | 52974 | 94.40 (93.63-95.16) |
